# Accuracy of ‘My Gut Feeling:’ Comparing System 1 to System 2 Decision-Making for Acuity Prediction, Disposition and Diagnosis in an Academic Emergency Department

**DOI:** 10.5811/westjem.2015.5.25301

**Published:** 2015-10-20

**Authors:** Daniel Cabrera, Jonathan F. Thomas, Jeffrey L. Wiswell, James M. Walston, Joel R. Anderson, Erik P. Hess, M. Fernanda Bellolio

**Affiliations:** Mayo Clinic College of Medicine, Department of Emergency Medicine, Rochester, Minnesota

## Abstract

**Introduction:**

Current cognitive sciences describe decision-making using the dual-process theory, where a System 1 is intuitive and a System 2 decision is hypothetico-deductive. We aim to compare the performance of these systems in determining patient acuity, disposition and diagnosis.

**Methods:**

Prospective observational study of emergency physicians assessing patients in the emergency department of an academic center. Physicians were provided the patient’s chief complaint and vital signs and allowed to observe the patient briefly. They were then asked to predict acuity, final disposition (home, intensive care unit (ICU), non-ICU bed) and diagnosis. A patient was classified as sick by the investigators using previously published objective criteria.

**Results:**

We obtained 662 observations from 289 patients. For acuity, the observers had a sensitivity of 73.9% (95% CI [67.7–79.5%]), specificity 83.3% (95% CI [79.5–86.7%]), positive predictive value 70.3% (95% CI [64.1–75.9%]) and negative predictive value 85.7% (95% CI [82.0–88.9%]). For final disposition, the observers made a correct prediction in 80.8% (95% CI [76.1–85.0%]) of the cases. For ICU admission, emergency physicians had a sensitivity of 33.9% (95% CI [22.1–47.4%]) and a specificity of 96.9% (95% CI [94.0–98.7%]). The correct diagnosis was made 54% of the time with the limited data available.

**Conclusion:**

System 1 decision-making based on limited information had a sensitivity close to 80% for acuity and disposition prediction, but the performance was lower for predicting ICU admission and diagnosis. System 1 decision-making appears insufficient for final decisions in these domains but likely provides a cognitive framework for System 2 decision-making.

## INTRODUCTION

During the last few decades, advances in cognitive science have significantly impacted our understanding of the cognitive aspects of bedside decision-making,^1^ particularly the observation of natural dual process behavior in clinical practice.^2^ Dual process theory illustrates a modulated interaction between a mainly intuitive system (System 1) and an idealistically-described hypothetico-deductive system (System 2).^3^ The first system, System 1, is rapid, automatic, almost completely unconscious, and requires minimal cognitive effort (your “gut feeling”). System 2, by comparison, is time and resource intensive, deliberate, requires significant cognitive effort, and is associated with hypothesis creation and testing.^4^

Clinical decision-making, particularly in emergency medicine (EM), exists in an environment of “bounded rationality” where there are significant constraints in regard to the information available, certainty, analytic time and available solutions.^5^ In this setting a skillful use of alternating System 1 and 2 decision processes can lead to efficient, economic and safe decision-making.^4^,^6^

Rapid recognition of a sick patient, along with fast and decisive decision-making, form the essence of EM.^7^ However, emergency physicians (EPs) treat patients with a spectrum of disease that varies from the entirely benign to the unstable, with often just a curtain or glass door separating the two. Regardless of severity, there is a mandate to provide high quality, safe and efficient care in the current medical environment.^8^

Although previous studies have addressed aspects of cognitive decision-making in daily practice,^6^ very few studies have described decision-making using the dual process theory^4^ framework and the performance and ultimate impact on patient care. A better understanding of the interaction of System 1 and 2 processes can lead to better quality decision making.^9^

We hypothesized that EPs are able to predict patient acuity (sick vs. not sick) and final disposition with a high degree of accuracy based on a limited amount of information using a System 1 process. We also sought to compare the accuracy of a provisional diagnosis based on a System 1 process and to the final diagnosis after the deliberative effect of System 2. Finally, we postulated that EPs’ performance in these domains improves with increasing experience and training.

## METHODS

This was a prospective observational study of a convenience sample of physicians enrolled during clinical shifts at different times of the day and evening, Monday through Sunday, from September–December 2013, including all acuity levels and chief complaints. The study was conducted in an academic emergency department with 73,000 annual patient visits that is certified as a Level 1 trauma center.

The study was approved and deemed exempt by the local institutional review board, as the participants in the study were physicians making clinical assessments, not patients. Prior to the start of the study, we wrote a detailed protocol and had a run-in period to refine the physician survey and standardized data abstract form. The lead investigator (D.C.) also trained the observers (J.F.T., J.R.A and J.M.W) in data acquisition.

EM board-certified attendings and EM residents [Post-graduate year 1 (PGY1) through PGY3] were eligible to be enrolled in this study and were asked to participate while working clinical shifts. A convenience sample of patients was assessed after being assigned to the care of the previously identified physicians; they were roomed in all areas of the emergency department (ED). The study was restricted to adult patients; we excluded patients transferred from an outside institution with an established diagnosis, a psychiatric complaint, known pregnancy, prisoners, patients in extremis (i.e. requiring emergent, life-saving interventions), and Level I and II trauma activations; otherwise, we included patients with all types of complaints (medical, orthopedic, minor trauma, gynecological, etc.) and well acuity levels.

As soon as a patient was roomed, a member of the study group identified the physicians assigned to care for the patient and administered a standardized survey. Physicians were provided and reviewed the first set of vital signs (often obtained by ambulance or by the triage nurse), the documented chief complaint, gender, age, and mode of arrival. Physicians were permitted to observe the patient for no more than 30 seconds. A brief greeting (e.g. “hello,” or “I will be right with you”) was also permitted to establish rapport.

With the limited information provided, we asked observer physicians to predict the following outcomes: 1) sick vs. not sick; 2) likely disposition (possibilities included dismissal home, ED observation unit, non-monitored hospital bed, telemetry bed and intensive care unit (ICU)); and 3) the likely diagnosis of the patient.

As there is no definition of sick widely accepted in the literature, we provided the observers the following working definition to cognitively frame their assessment: “A patient is sick when he/she has a condition that, when left undiagnosed or untreated, may develop into a life or limb threat or cause disability.”

One week after the index ED presentation, we assessed the clinical records of enrolled patients to evaluate outcomes and obtain follow-up data. For the variable sick vs. not-sick, we used and adapted previously published^4^ objective criteria that include discrete procedure (e.g., intubation), outcomes (e.g., admission to an ICU), administrative data (e.g., critical care time billing) and commonly-accepted diseases processes associated with high acuity in the ED (Appendix). Two authors (J.F.T. and J.L.W.) reviewed each sick/not sick prediction and compared it to defined criteria to ascertain if the prediction was correct or not; when disagreement existed, the lead author adjudicated the classification (D.C.). Agreement between observes was calculated using Cohen’s kappa coefficient.

For the variable of disposition, we grouped the responses into three categories to facilitate analysis: 1) dismissal, 2) admission to a non-ICU unit (ED observation unit, regular floor and telemetry), and 3) ICU. Two authors reviewed the disposition prediction and compared it to the final disposition.

For the variable diagnosis, two authors reviewed each predicted diagnosis and compared it either to the final ED diagnosis, bounce back within 72h diagnosis or final hospital diagnosis, using that order of hierarchy. If disagreement arose, the lead author adjudicated the outcome classification. Agreement between observers was calculated using Cohen’s kappa coefficient.

We took the following steps to reduce the risk bias in our study: (1) determined inclusion and exclusion criteria prior to data collection and analysis; (2) calculated power and sample size prior to the conducting the study; (3) developed and piloted a standardized data collection form before use in the study; (4) ensured all the patients had similar probability of selection as enrollment depended of the time of the day and not on patient characteristics (although we did enroll a convenience sample); (5) did not blind observers and data collectors to the study objectives and hypothesis (however, the verbal responses of the physicians did not depend on the judgment of study personnel); (6) performed a prospective study, so outcomes had not occurred at the time of data collection; (8) arranged for the data collectors to meet periodically with the primary investigator to review questions; (9) calculated inter-rater reliability and agreement for the outcome variables “sick” vs. not sick” and “final diagnosis;” and (10) discussed disagreements with the primary investigator who adjudicated outcome classifications.

Based on our previous published article,^4^ we calculated power and samples size with an estimated difference of acuity of 15% and a sensitivity for attending physicians of 80%. We estimated that in order to detect meaningful differences between EM attendings and residents, we needed a total of 390 observations, two-thirds from the resident physicians and one-third from the attending physicians. The observed difference in acuity prediction sensitivity between attendings and residents was less than 6%.

We tabulated data in a Microsoft Excel spreadsheet, and statistical analyses were conducted using JMP software version 9.0, (S.A.S. Institute, Chicago). For normally distributed variables, we calculated mean and standard deviations (SD) and used parametric tests; for skewed data, median and interquartile ranges were reported and non-parametric tests were applied. We constructed two-by-two contingency tables to calculate prognostic performance estimates. We assessed sensitivity, specificity, likelihood ratios, positive and negative predictive values (PPV and NPV), and obtained 95% confidence intervals (CI) using Meta-DiSc software.^10^ A statistician not involved in the study calculated power and sample size of the protocol and reviewed all data procedures and analyses.

## RESULTS

We collected 662 observations from 289 patients. Among the 662 observations, 417 (63%) were performed by residents (PGY1 16%, PGY2 20% and PGY3 27%) and 245 (37%) by attendings. The rates of admission of acuity of the patients were similar to the historic data available for the department.

Participating physicians classified 37% (242) of the patients as sick, while the investigators classified 34.3% as fulfilling the sick definition. Inter-observer agreement between the two investigators applying the sick definition had a kappa of 0.97 (95% CI [0.95–0.99], p<0.0001).

For the sick vs. not-sick variable, physicians had an overall sensitivity of 73.9% (95% CI [67.7%–79.5%]), specificity of 83.3% (95% CI [79.5%–86.7%]), PPV of 70.3% and NPV of 85.7% when compared to the gold standard definition of sick (Table 1). Attendings had a sensitivity of 77.5% (95% CI [66.8–86.1%]), specificity of 83.1% (95% CI [76.6–88.5%]), whereas residents had a sensitivity of 72.0% (95% CI [64.1–79.0%]) and specificity of 83.5% (95% CI [78.4–87.7%]). The difference in sensitivity between attending and resident physicians was not statistically significant.

For the disposition variable (discharge versus hospital admission), 50.4% of patients were admitted, physicians overall had a sensitivity of 80.8% (95% CI [76.1–85.0%]) and a specificity of 75.3% (95% CI [70.4–79.8%]) (Table 2). Of the admitted patients 18% required an ICU bed; when analyzing admissions to ICU vs. non-ICU, the overall sensitivity was 33.9% (95% CI [22.1–47.4%]) and a specificity was 96.9% (94.0 to 98.7%). When comparing the performance between attending and resident physicians, attendings had a sensitivity of 42.9% (95% CI [21.9–66.0%]), specificity of 96.7% (95% CI [90.7–99.3%]), PPV of 75% (95% CI [42.8–94.2%]) and NPV of 88% (95% CI [80.0–93.6%]). Residents had a sensitivity of 29%, specificity of 97%, PPV of 68% and NPV 85%. The difference in performance between attending and resident physicians was not statistically significant.

Finally, for the diagnosis variable; the predicted diagnosis compared to the final diagnosis (ED final diagnosis, 72-hour bounceback diagnosis or hospital final diagnosis) was correct in 54% of the patients, 56.9% for attendings and 52.2% with no statistical difference (p=0.24) for residents. Inter-observer agreement between investigators had a kappa 0.91 (95% CI [0.87–0.94], p<0.0001). Attendings were able to predict the diagnosis correctly in 53.9% of the cases, while the residents were accurate 52.2% of the time. The difference in performance between attending and resident physicians was not statistically significant.

When analyzing vital signs we found that patients in the “sick” category had a higher median (IQR) temperature [36.7 (36.6–36.9) vs. 37.0 (36.6–37.3), p<0.0001]; higher mean (SD) heart rate [81.4 (16.6) vs. 90.1 (25.8), p<0.0001]; lower diastolic blood pressure [79.3 (14.9) vs. 74.5 (19.5), p=0.0005]; increased mean (SD) respiratory rate [17.1 (2.8) vs. 18.4 (5.6), p<0.0001] and a lower median (IQR) SO_2_ [98 (96–99) vs. 97 (IQR 95–99), p=0.012] than not-sick patients.

## LIMITATIONS

The dual process-theory model^3^,^11^ is not a universally accepted paradigm to explain clinical decision-making. Although it is widely used and considered valid in EM,^1^ some have challenged the usefulness and validity of the model^12^ and proposed that an intertwined dichotomic approach cannot be observed in all aspects of decision-making. The nature of decision-making lies between the task itself and the mental model of the person performing the decision; it is likely that some decisions cannot be classified as belonging to System 1 or 2 and may be more appropriately described as quasi-rational.^12^–^13^

There is no universally accepted definition of “sick” in the scientific literature. We developed a definition of sick based on financial, operational and educational rationale to classify the outcomes, adapting criteria used in previous literature.^4^ Given the ambiguity of the concept, we attempted to provide the observers with a cognitive framework and gave them an a priori definition of “sick” when conducting the study.

Another limitation, bounded by this naturalistic approach, is the potential bias that asking observers to make a prediction may introduce. Asking observers to provide a prediction based on limited information may inappropriately anchor the observer, such that System 2 is subsequently unable to override System 1 decision-making process.^14^ A possible study design involving a third non-clinically-related party making the sick vs. not-sick judgment although free of this bias will also be free of the environmental cognitive factors that affect decision making in a real-life scenario.

This study attempted to naturalistically observe real-time, clinical task performance in a very information- constrained System 1 decision-making model as it pertains to evaluation in the emergency setting. Although the literature has previous studies about the real-life performance of complex decision making, few studies^4^ have been able to assess this process bounded by clinical constraints and this represent the most important strength of this study.

## DISCUSSION

Physicians’ performance using System 1 reasoning to predict acuity (i.e., sick vs. not-sick) had sensitivity of 73.9% and specificity of 83.3%. In terms of disposition prediction, performance was similar to the acuity prediction, with a sensitivity of 80.8% and specificity of 85.3%. This performance results in a positive likelihood ratio (+LR) of 4.4 and a negative likelihood ratio (−LR) of 0.31; the performance of the prediction for the disposition prediction yield a +LR of 3.27 and −LR of 0.25, while for the ICU vs. non-ICU yield a +LR of 11 and −LR 0.68. These test characteristics offer a favorable profile significantly improving the post-test probability of patients deemed to be sick by the observer and help predict disposition accurately. We observed no statistically significant difference between attendings and residents. Finally, the predictive accuracy for diagnosis was 53.9% overall; this is quite low and likely does not permit physicians to make definitive diagnoses solely based on a System 1 process alone.

This study had slightly different methodology compared to previous studies.^4^,^6^ This time we provided the physicians with a short operational definition of the meaning of sick; we believe this represents an improvement in the methodology as it provided a clearer cognitive framework for the prediction. Another difference from previous studies was a larger observation collection, which we believe made the results more robust.^4^

## CONCLUSION

The overall performance of nearly 80% sensitivity with a +LR of 4.4 for acuity appears to be appropriate given the limited information provided, but it is not powerful enough to make a final acuity assessment on these patients. System 1, however, appears to be appropriate to provide a cognitive framework for the later System 2 dysrationalia override.^1^,^11^ Correctly predicting the disposition and acuity in four of every five patients, with +LR between 3.27 for admission and a very powerful +LR of 11 for ICU admission, appears to be appropriate enough to start a working disposition and evaluation while refining the overall clinical hypothesis.

Emergency medicine is defined by timely and accurate decision-making and the initiation of life-, limb-, or eyesight-saving interventions.^4^,^7^ In an ideal scenario, the healthcare team should have sufficient time, information and resources to make the best possible decision regarding a patient. However, our decision-making is not truly rational, as not every single possible decision is considered and is bounded by the constraints of available resources.^5^ Albeit far from a very accurate prediction power, the performance of System 1 reasoning appears to be adequate to provide a cognitive framework to enable emergency physicians to determine a provisional diagnosis, initiate early interventions, and make disposition decisions when resource are limited. However, this reasoning requires System 2 refinement later in the encounter to ensure the delivery of high quality care.

## Figures and Tables

**Table 1:** Performance of the prediction of sick vs. not-sick patients by emergency physicians.

	All physicians (95% CI)	Attendings (95% CI)	Residents (95% CI)
Sensitivity	73.9% (67.7 to 79.5%)	77.5% (66.8 to 86.1%)	72.0% (64.1 to 79.0%)
Specificity	83.3% (79.5 to 86.7%)	83.1% (76.6 to 88.5%)	83.5% (78.4 to 87.7%)
PPV	70.3% (64.1 to 75.9%)	68.9% (58.3 to 78.2%)	71.1% (63.2 to 78.1%)
NPV	85.7% (82.0 to 88.9%)	88.5% (82.4 to 93.0%)	84.1% (79.1 to 88.3%)

*PPV,* positive predictive value; *NPV,* negative predictive value

**Table 2:** Performance of the prediction of disposition.

	All physicians (95% CI)	Attendings (95% CI)	Residents (95% CI)
Dismissal vs. admission			
Sensitivity	80.8% (76.1 to 85.0%)	80.4% (71.8 to 87.3%)	81.1% (75.0 to 86.2%)
Specificity	75.3% (70.4 to 79.8%)	80.6%(72.9 to 86.9%)	71.9% (65.3 to 77.9%)
PPV	75.2% (70.2 to 79.6%)	77.6% (68.9 to 84.8%)	73.9% (67.7 to 79.5%)
NPV	80.9% (76.2 to 85.1%)	83.1% (75.5 to 89.1%)	79.5% (73.0 to 85.0%)
ICU vs. non-ICU admission			
Sensitivity	33.9% (22.1 to 47.4%)	42.9% (21.9 to 66.0%)	29.0% (15.4 to 45.9%)
Specificity	96.9% (94.0 to 98.7%)	96.7% (90.7 to 99.3%)	97.0% (93.2 to 99.0%)
PPV	71.4% (51.3 to 86.7%)	75.0% (42.8 to 94.2%)	68.8% (41.4 to 88.9%)
NPV	86.6% (82.1 to 90.3%)	88.0% (80.0 to 93.6%)	85.8% (80.0 to 90.4%)

*PPV,* positive predictive value; *NPV,* negative predictive value; *ICU,* intensive care unit

## References

[1] Croskerry P (2009). A universal model of diagnostic reasoning. Acad Med.

[2] Croskerry P, Petrie DA, Reilly JB (2014). Deciding About Fast and Slow Decisions. Acad Med.

[3] Sloman SA (1996). The empirical case for two systems of reasoning. Psychol Bull.

[4] Wiswell J, Tsao K, Bellolio MF (2013). “Sick” or “not-sick”: accuracy of System 1 diagnostic reasoning for the prediction of disposition and acuity in patients presenting to an academic ED. Am J Emerg Med.

[5] March JG (1994). Chapter 1 Limited rationality. Primer on Decision Making: How Decisions Happen.

[6] Calder LA, Forster AJ, Stiell IG (2012). Mapping Out the Emergency Department Disposition Decision for High-Acuity Patients. Ann Emerg Med.

[7] Rosen P (1979). The biology of emergency medicine. JACEP.

[8] Graff L, Stevens C, Spaite D (2002). Measuring and improving quality in emergency medicine. Acad Emerg Med Off J Soc Acad Emerg Med.

[9] Croskerry P (2000). The Cognitive Imperative Thinking about How We Think. Acad Emerg Med.

[10] Zamora J, Abraira V, Muriel A (2006). Meta-DiSc: a software for meta-analysis of test accuracy data. BMC Med Res Methodol.

[11] Sloman SA, Gilovich T, Griffin D, Kahneman D (2002). Two systems of reasoning. Heuristics and Biases: The Psychology of Intuitive Judgment.

[12] Custers EJFM (2013). Medical Education and Cognitive Continuum Theory: An Alternative Perspective on Medical Problem Solving and Clinical Reasoning. Acad Med.

[13] Hammond KR (2010). Intuition, No! …Quasirationality, Yes!. Psychol Inq.

[14] Kahneman D, Part I (2011). Two systems. Thinking, Fast and Slow.

