# Supplementary material for: Accuracy of ‘My Gut Feeling:’ Comparing System 1 to System 2 Decision-Making for Acuity Prediction, Disposition and Diagnosis in an Academic Emergency Department
Source: West J Emerg Med. 2015 Oct 20;16(5):653–7. doi: 10.5811/westjem.2015.5.25301 (PMC4644030; doi:10.5811/westjem.2015.5.25301)
Supplement: Supplementary file 1 [file wjem-16-653-s001.pdf]

Appendix 1. Conditions defining Sick.

Disposition

- Directly to or
- ICU admit
- Telemetry admit
- Death
- Hospice initiation

Cardiac

- ACS if admitted with diagnosis of ACS; positive cardiac markers or emergent catheterization
- Symptomatic arrhythmia
- Decompensated CHF/pulmonary edema
- Symptomatic pericardial effusion/tamponade
- Hypertensive emergency requiring iv vasoactive medications

Dermatology

- Steven Johnson Syndrome
- Toxic Epidermal Necrolysis
- Staphylococcal scalded skin syndrome

Drug-related

- Overdose with end-organ damage or abnormal vital signs
- Agitated delirium
- Medication side effect with end-organ damage or abnormal vital signs
- Anaphylaxis
- Angioedema
- Naloxone administration
- Epinephrine required

Electrolyte

- Potassium below 2.5 or greater than 6.0

- Sodium below 125 or greater than 150 requiring treatment
- Hypo/hypercalcemia requiring treatment

Endocrine

- Thyroid storm/myxedema coma
- Adrenal insufficiency/collapse
- Hyper/hypocalcemia
- Diabetic ketoacidosis

ENT

- Tracheo-innominate artery fistula
- Malignant otitis externa
- Mucormycosis

Environmental

- Hypo/hyperthermia
- Electrocution
- Envenomation
- Rabies
- Toxic exposure with end-organ damage or abnormal vital signs
- Carbon monoxide poisoning
- Methemoglobinemia
- Cyanide poisoning

Abdominal

- Gastrointestinal bleed (upper/lower) if requiring IV fluids or blood transfusion
- Cholangitis
- Pancreatitis
- Liver failure/hepatitis

Hematologic

- Bleeding and coagulopathy requiring transfusion and/or anticoagulant reversal

- Thrombotic thrombocytopenic purpura
- Disseminated intravascular coagulation
- Symptomatic anemia

Infectious diseases

- Sepsis
- Bacteremia
- Viremia
- Meningitis
- Encephalitis
- Necrotizing fasciitis
- Toxic shock syndrome
- Aids defining disease process
- Acute hepatitis

Nephrology

- Emergent dialysis
- Acute kidney injury
- Rhabdomyolysis

Neoplastic

- New diagnosis leukemia/lymphoma
- Tumor lysis syndrome
- Neutropenic fever

Neurology

- Intra-cerebral hemorrhage
- Stroke
- Guillain-barre syndrome
- Cord compression syndromes
- Acute paralysis

- Myasthenia crisis
- Status epilepticus
- Acute intracranial hemorrhage
- Transient ischemic accident
- Altered mental status secondary to systemic infection
- Movement disorder
- Intra-cerebral hemorrhage, trauma or non-trauma
- Traumatic brain injury
- Intracranial abscess

Orthopedics

- Unstable spine fracture
- Fracture with neurovascular compromise
- Grade IIIA-C open fracture
- Septic joint
- Flexor tenosynovitis
- Compartment syndrome
- Dislocation of major joint
- Long bone fracture

Procedure

- Intubation
- Central line placement
- Chest tube
- Pericardiocentesis

Pulmonary

- Respiratory failure
- Refractory asthma exacerbation
- Pulmonary embolism

- Massive hemoptysis
- Pneumothorax/hemothorax

Surgical

- Bowel perforation
- Uncontrolled hemorrhage requiring operation/embolization
- Esophageal perforation
- Level 3 trauma injury requiring urgent/immediate operation or ICU stay
- Appendicitis
- Complicated diverticulitis
- Intra-abdominal abscess
- Volvulus
- Intra-abdominal hemorrhage
- Bowel obstruction small or large
- Symptomatic/ruptured aaa
- Mesenteric ischemia
- Pulseless extremity

Urology

- Testicular torsion
- Urethral obstruction
- Priapism
- Infected kidney stone
- Perinephric abscess
